# International Case Studies to Identify Success Factors and Contextual Conditions in the Digital Transformation of Health Care Systems and Derive Lessons for Germany: Study Protocol for a Mixed Methods Study

**DOI:** 10.2196/80301

**Published:** 2026-01-21

**Authors:** Lena Kraft, Anna-Lena Brecher, Sophia Sgraja, Reinhard Busse, Volker Eric Amelung

**Affiliations:** 1 Hannover Medical School, Institute for Epidemiology, Social Medicine, and Health Systems Research, Carl-Neuberg-Straße 1, Hannover, 30625, Germany, 495115320; 2 Department of Health Care Management, Technische Universität Berlin, Berlin, Germany

**Keywords:** digital health, international health care systems, case study research, success factors, tipping points

## Abstract

**Background:**

Germany’s health care system continues to face significant challenges in its digital transformation due to outdated structures, interoperability issues, strict data protection regulations, and low user acceptance, despite numerous legislative initiatives, such as the Digital Care Act in 2019, which was intended to promote practical use and innovation. In contrast, several international health care systems have successfully advanced their digital transformation, offering valuable insights and potential lessons for the German health care system.

**Objective:**

This study, as part of the research project “NADI: Benefits and Acceptance of Digital Health,” analyzes international health care systems to identify key success factors and develop pragmatic recommendations for German policymakers to enhance the country’s digital health implementation.

**Methods:**

This study uses a mixed methods triangulation approach, combining case study selection, qualitative expert interviews, and a quantitative online survey to develop actionable policy recommendations for the digital transformation of health care in Germany. The study applies the conceptual framework of tipping points and success factors to identify critical factors in the digital transformation of health care systems, where certain actions or conditions fundamentally influence adoption and success. A total of more than 100 interviews were conducted with experts representing 8 stakeholder groups from 9 different health care systems. The qualitative data are evaluated using qualitative content analysis according to Kuckartz and Rädiker. In an online survey, a minimum of 305 participants from the German health care system will be surveyed regarding the relevance and feasibility of the key success factors identified in the international case studies. The dataset will be analyzed statistically using SPSS, both descriptively and inferentially (eg, subgroup analyses).

**Results:**

Between November 2024 and September 2025, interviews with international health care experts were conducted. As of October 2025, the qualitative content analysis is still ongoing. The recruitment phase for the online survey is planned from October 15 to December 15, 2025. Initial results are expected to be available in 2026. The study protocol was submitted during the qualitative data collection phase before the commencement of the quantitative survey. Analysis had not yet begun at the time of submission.

**Conclusions:**

The use of a case study methodology has been demonstrated to facilitate the acquisition of invaluable insights into international best practices, while concurrently offering the opportunity to identify specific success and failure factors. The integration of qualitative expert interviews serves to contextualize international findings on tipping points and success factors in the implementation and use of digital health tools. The transfer of the international results to the German context represents a central component of the research project, which aims to investigate practical implementation. The combination of these approaches forms a comprehensive basis for deriving specific recommendations for action for the German health care system.

## Introduction

Germany is encountering challenges in its digital transformation, particularly in the health care sector. For instance, the electronic patient file was legally integrated into the German Social Code (Sozialgesetzbuch) as early as 2004 [1]. This was followed by a period of 20 years characterized by numerous legislative initiatives, such as the resolutions on the E-Health Act in 2015 to expand the telematics infrastructure or the Digital Care Act in 2019, which was intended to promote the practical use and innovation of digital health technologies [1,2]. It was not until March 2024 that the Act to Accelerate the Healthcare System came into force as part of the Federal Ministry of Health’s digitalization strategy, in which the establishment of the electronic health record as a central component is now being driven forward [2]. Thus, there was clearly no shortage of political initiatives [3]. Rather, the reasons for the weak implementation of digital health in Germany seem to be attributable to a number of factors, including the heterogeneity of information technology (IT) systems and the lack of interoperability [4], which are exacerbated by the fragmented health care system in Germany. In addition, significant public concerns regarding unauthorized data access and data protection issues are holding back the digital transformation [5]. Moreover, a dearth of acceptance among users, such as patients or physicians, is frequently attributable to a paucity of skills [3].

Other European countries, including Denmark and Estonia, as well as countries in more distant regions, such as Israel, have demonstrated a high level of development in digital structures in the health care sector compared to Germany and face fewer challenges in the areas described above and beyond [6]. For example, Estonia and Denmark are focusing on a central platform for health data, ease of use, and public trust in digital health tools [7]. Israel, on the other hand, is strongly committed to start-ups through the provision of grants, tax incentives, and favorable regulations [8]. Nevertheless, the digital transformation within the German health care system is already underway. Electronic health cards, electronic prescriptions, and reimbursable digital health applications, among other digital components, have been implemented and are being used to varying degrees [9]. To exploit the full potential of this transformation, the right framework conditions are needed, such as education and incentive mechanisms. It is important to identify success factors in this context to improve conditions and accelerate the implementation and use of digital tools.

This study constitutes a component of the research project *NADI: Benefits and Acceptance of Digital Health*, which is funded by the German Innovation Fund. The project involves the analysis of international experiences, options for action, and patient preferences. This part of the study aims to identify success and failure factors, as well as tipping points, in the digital transformation of international health care systems. By examining a selection of international case studies and best practices, this study will uncover insights into what contributes to successful digitalization in health care, for example, success factors with regard to regulation or financing. The core findings will then be evaluated for their transferability to the German health care system. Considering the findings from all project components of the other project partners, a series of pragmatic recommendations is set to be developed. These recommendations are designed to assist German policymakers in effectively overseeing the ongoing transformation toward digital health. Furthermore, these recommendations are intended to ensure that the requisite measures are implemented to ensure a successful and sustainable digitalization process.

The aim of this study is therefore to identify success and failure factors in the process of digitalization of health care systems and to analyze these in the context of the individual conditions and structures of the respective health care system. Finally, recommendations for action to promote digital transformation in the German health care system will be derived. The focus is not on specific technological innovations or applications but rather on the political and sociocultural processes that have set the course for successful digitalization in health care, adopting a rather broad perspective on digital health. The subsequent research questions are as follows.

- What are the relevant success and failure factors in the digital transformation of health care systems?
- What are the relevant tipping points in the digital transformation of health care systems?
- Which success factors from other countries in the digital transformation of health care systems can be transferred into recommendations for action for the German health care system?

## Methods

### Study Design

**Figure 1.**
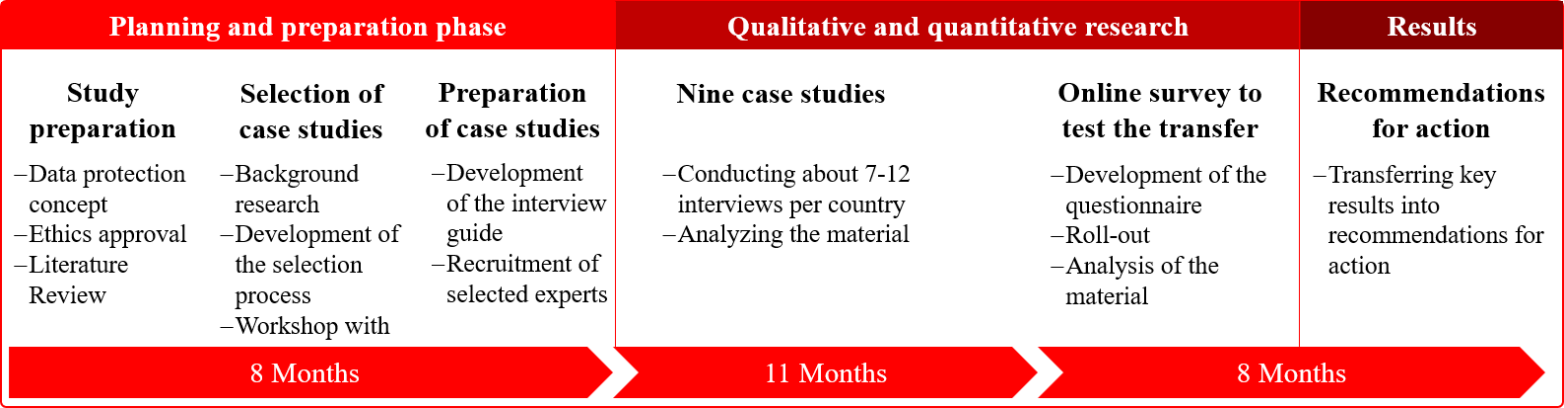
Study design. This figure shows the flowchart of the project process in 2024-2026.

The study follows a mixed methods triangulation approach. It includes case studies, comprising extensive literature research and semistructured expert interviews, as well as a quantitative online survey. In a concluding stage, the results of the analysis of the interviews and the quantitative data will inform the development of practical recommendations and measures for health policy regarding the digital transformation of the German health care system (Figure 1). The study was developed with the support of the scientific advisory board. Patients were not involved in the design, development, or dissemination plans of the research.

### Success Factor Research

The research of success factors constitutes a subdiscipline of business administration. Its purpose is to identify the factors that have contributed positively or negatively to the success of a company. According to Steiner [10], “Strategic factors refer to an action, element, or condition which for a business may be of critical importance in its success or failure.” In this study, we will apply success factor research to a different research discipline, in which the focus is not on individual companies but on entire health care systems [10]. Success in this case is the successful implementation of key digital health tools, characterized by a high application rate and user acceptance, as well as a positive impact of the tools on care and communication processes in the health care system.

### Theory of Tipping Points

Another theory that is being addressed as part of this research project is the concept of tipping points. This theory was popularized by the book *The Tipping Point* by Malcolm Gladwell, first published in 2000 [11]. Since then, it has been increasingly used in many different research disciplines [12,13]. The tipping point is defined by Gladwell [11] as “that magic moment when an idea, trend, or social behavior crosses a threshold, tips, and spreads like wildfire.” In this study, the concept of tipping points is used to identify critical phases in which the digital transformation in the health care systems examined has been fundamentally influenced. Triggers could include certain actions, such as political strategies, the implementation of particular technologies, legal changes, adjustments to provider remuneration, or public campaigns, as well as overarching conditions, such as changes in governance or the occurrence of a pandemic. Recognizing these pivotal moments is crucial to identifying the catalysts and analyzing them as potential success factors.

### Case Studies

To identify success factors and tipping points of digital health transformation, it is essential to consider the specific context, for example, the conditions of the respective health care system. According to Yin [14], a case can be defined as “a contemporary phenomenon within its real-life context, especially when the boundaries between a phenomenon and context are not clear and the researcher has little control over the phenomenon and the context”. Therefore, the case study approach is well suited to systematically gathering international experiences regarding the digitalization of health care systems.

This study uses an exploratory case study approach, which is 1 of 3 possible approaches. However, the boundaries between descriptive, explanatory, and exploratory research are flexible and overlap to some extent [15]. In addition to Yin, Merriam and Stake [15,16] have particularly shaped and further developed the case study research approach. The approach applied in this study is primarily based on the recommendations of Merriam, which are associated with the constructivist case study paradigm. In accordance with Merriam’s recommendations, the case study design and research questions are based on a literature review. The qualitative data collection for this study is based on interviews and the analysis of documents. As Merriam describes it, the analysis of the data is “the process of making meaning” [11] and is approached through qualitative content analysis [9]. Merriam further recommends various strategies for validating the results of case study research in terms of internal and external validity, such as triangulation and exchange with experts, as well as a very comprehensive description of the cases, as is also implemented in the context of this study [9,10].

The following section delineates the steps involved in conducting the case studies and the planned evaluation.

### Case Study Selection

**Figure 2.**
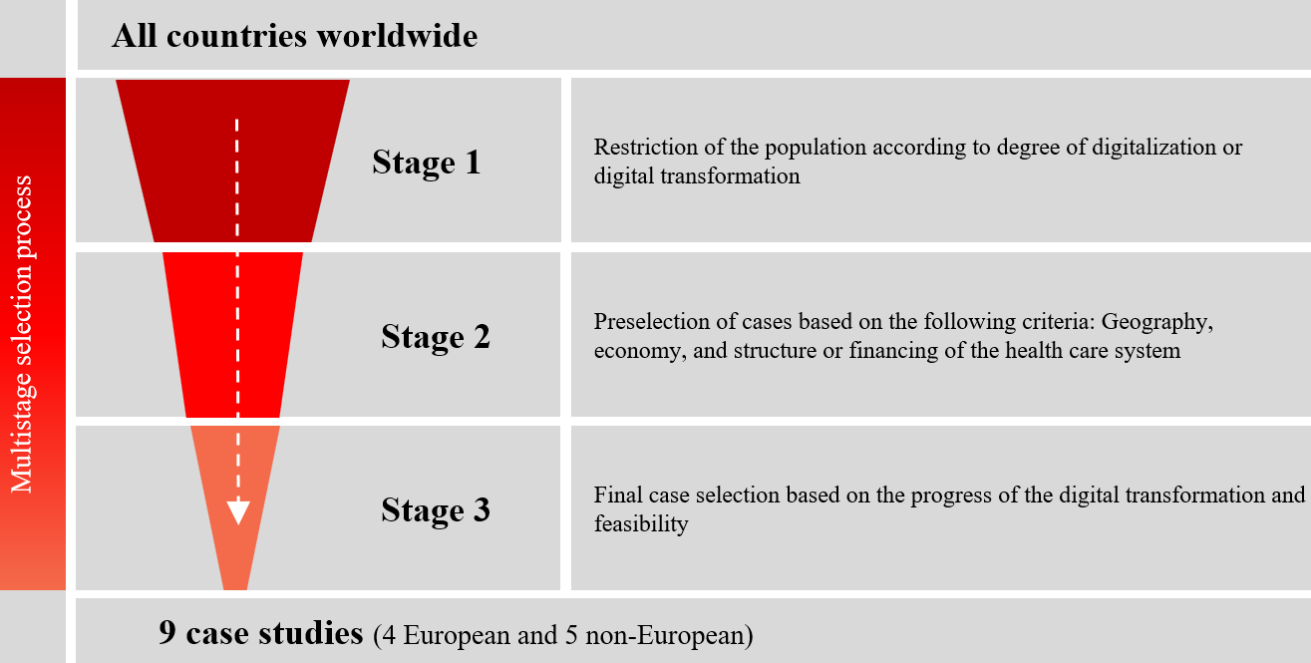
Case study selection. This figure shows the 3-stage process of case study selection.

The selection of countries for case studies is often perceived as arbitrary, and there is currently insufficient research on this approach. Nevertheless, a reasoned and transparent approach is necessary to reduce bias in case selection and to maximize knowledge gain [17]. In this study, 9 international case studies were identified through a multistage, criteria-driven process (Figure 2). The study uses the method of diverse case study selection, which is particularly recommended for exploratory research approaches. An inherent limitation of the case study approach is its lack of representativeness. Nevertheless, the selection of cases that demonstrate a wide range of characteristics with respect to relevant variables can enhance representativeness by reflecting the diversity of the overall pool [12].

The selection process for the case study countries was conducted in 3 stages, following an iterative approach. The first stage involves narrowing down the initial pool, encompassing all health care systems worldwide, based on their degree of digitalization, thereby including both countries with well-established digital health systems and those that have undergone significant digital transformation in recent years. To assess the level of digitalization, several international indices were consulted, including the World Digital Competitiveness Ranking 2023, the Global Digital Health Monitor (latest update in 2023), the Digital Economy and Society Index 2022, the Digital Riser Reports 2020 and 2021, and the Bertelsmann Foundation’s 2018 study on international health care system comparisons with a focus on digitalization [6,18-22]. Relevant indicators include strategy and policy, workforce, standards and interoperability, mindset, and infrastructure. Additionally, expert opinions from the project’s scientific advisory board were incorporated to account for recent developments in the digitalization of various health care systems.

In the second stage, a set of relevant criteria was selected to characterize the countries in the preliminary selection. These criteria were grouped into three overarching categories: (1) geography, (2) economy, and (3) health care system (Table 1).

This structured overview aimed to facilitate the selection of a diverse set of case studies. The goal was to ensure a broad spectrum of insights while maximizing the relevance and transferability of findings to the German health care system. The characterization of a short list of countries included in stage 2 can be found in Multimedia Appendix 1.

**Figure 3.**
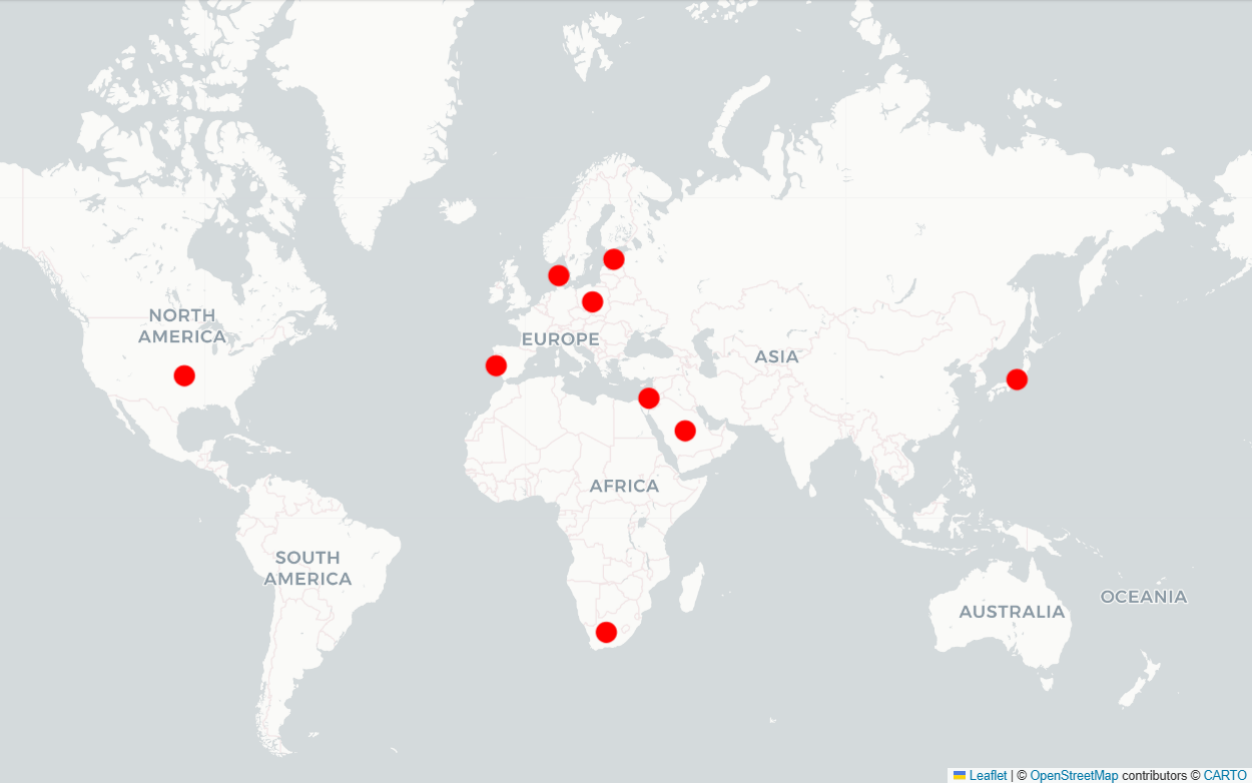
Map of selected case studies. This figure illustrates the selected case studies on a world map. Source: The authors’ illustration created using Python code and the Folium library (Copyright 2013–2025 Rob Story) [25]. Geospatial data were obtained from OpenStreetMap (Copyright OpenStreetMap contributors; data licensed under the Open Database License version 1.0) [26].

The final selection of countries in stage 3 was based on an in-depth assessment of a short list of countries identified as suitable in the previous stages. This assessment particularly considered the actual state of health care digitalization and recent developments in the implementation of key digital health tools. In addition, practical considerations, including access to scientific literature, availability of experts, political stability, and the logistical and financial feasibility of conducting the interviews on site, were taken into account. The selection process was further refined through expert discussions and insights gained from semistructured expert interviews. The 9 selected case studies are as follows (Figure 3): Estonia, Denmark, Poland, and Portugal (European countries); Israel, Japan, Saudi Arabia, South Africa, and the United States (specifically the United States Department of Veterans Affairs; non-European countries).

laGiven the complexity of the health care system in the United States, this case study will focus on the Veterans Affairs system, the largest integrated health care system in the country, providing universal health care to approximately 9.1 million eligible veterans [23,24].

**Table 1.** Case study characterization criteria. This table shows the criteria that were considered in stage 2 of the selection of the case studies.

Category and criteria	Indicators
Geography	
Geographic region	WHO^a^ regions
Population	Number of inhabitants
Area	Area in km^2^
Economy	
Economic development	High-income/low-income countries
Economic performance	GDP^b^ in US $ per capita
Health care system	
Health care system typology	National health service/social insurance/private insurance
Centralization in health care governance	Centralized/decentralized
Health care expenditure	In US $ per capita

a WHO: World Health Organization

b GDP: Gross Domestic Product.

### Qualitative Survey

#### Background Research

Following the selection of case studies, a comprehensive background research phase was conducted. This phase focuses on characterizing the health care system of each selected country, identifying key institutions involved in health care delivery, and assessing the development and current state of digitalization in the health care sector. The research also identified relevant stakeholders, such as government agencies, health care providers, technology companies, and patient organizations, to understand their roles in the digital transformation process. This background information provides essential context for the case studies, ensuring a thorough understanding of the health care systems and their digitalization trajectories before conducting the interviews.

#### Conception of the Expert Interview

The conceptualization and planned reporting of the qualitative data collection are grounded in the COREQ (“Consolidated Criteria for Reporting Qualitative Research”) guidelines (Checklist 1), thereby ensuring a systematic approach [19]. As the analysis is ongoing, not all the criteria in the checklist have been met yet. To generate specific information on the digital transformation in the selected countries, it was decided to conduct expert interviews. People involved in the context of digital health and who have relevant specialist knowledge are designated as experts. If the individuals have specific role knowledge or can be considered to have special expertise in the field of digital health, these experts are consulted. Using Helfferich’s methodology, a structured framework of a semistructured interview guide was devised for the interviews with these experts, focusing on factual questions to elicit their expertise [20]. This approach ensures the collection of pertinent role-specific knowledge. In accordance with this methodology, interview sections are thematically constrained to enable experts to efficiently reproduce their knowledge, given the limited time available. Experts are permitted to respond in detail to specific domains of knowledge or to provide concise statements. The interviewer is assisted by the semistructured interview guide to present themselves professionally and competently, thereby reducing the power imbalance between interviewer and interviewee [20].

#### Development of the Semistructured Interview Guide

The structure of the interview guide was developed in accordance with the requirements and principles of guide development as outlined by Helfferich and Kruse [27,28]. The design of the semistructured interview guide was informed by these principles, with the objective of addressing the research interest in an appropriate manner, while maintaining a high degree of openness and structure. The 4 steps of Helfferich’s procedure were used as a framework, encompassing collection, verification, organization, and integration [27].

In the initial step, a range of questions was compiled, which was then reviewed in the subsequent step with respect to their expressive potential and relevance in addressing the research queries. In the third step, pertinent questions were categorized according to their content. In addition, overarching modules and subcategories were formulated in this step. In the final step, narrative-generating impulses were assigned to the developed modules. The content of the semistructured interview guide is based on findings from previous research and workshops with the project’s advisory board. The following categories have been developed and will guide the interviewer and interviewee through the interview: (1) tipping points, (2) governance, (3) incentives and sanctions, (4) technical regulations and structures of interoperability, (5) digital health tools, and (6) recommended actions.

In the inaugural module of tipping points, the expert is queried on the events that have precipitated the digital health transformation in the respective nation. This is followed by inquiries into the influence of the health system’s structure on the transformation, incentive mechanisms, and potential sanctions. Additionally, questions regarding technical infrastructure and interoperability are posed. Ultimately, the implementation of digital health tools, including diagnosis-, system-, or prevention-oriented tools and tools for collaboration with other health care providers, is subjected to scrutiny. Finally, the conversation is concluded with a question regarding the key lessons for the digital transformation of a health care system. A series of documents was created for each country, with adaptations made to align with the stakeholder groups and the 4 distinct tools. This approach was adopted to ensure the collection of more precise and detailed information. However, to maintain the comparability of the interviews, the structure of the interview guide remained unaltered, with only minor adjustments made to the content. The organization of pertinent questions, the development of modules, and the allocation of narrative-generating prompts were subjects of repeated deliberations with other researchers, leading to successive revisions [27,28]. The basic interview guide for the interviews was subjected to rigorous testing in accordance with the prevailing standards of scientific research [29,30]. Initially, the semistructured interview guide was submitted for independent evaluation to 2 experienced methodologists specializing in qualitative research. Concurrently, 4 pretest remote interviews were conducted with experts from Portugal and Poland to assess the efficacy of the semistructured guide in a field setting. The comments of the 2 experts in qualitative methods and the experiences from the 4 pretest interviews were discussed and reflected upon by the project team, and the interview guide was revised and finalized accordingly. The semistructured interview guide can be found in Multimedia Appendix 2.

#### Recruitment of the Interviewees

Participants for the case studies were identified through background research for each country and were sought from the following areas: health care payers, service provision (outpatient and inpatient care), IT and industry sector, patient representatives, science, and other categories (such as nonprofit organizations).

The basis for the inclusion of suitable participants was predicated on digital health expertise. Further interviews were conducted until theoretical saturation was achieved, as described by Glaser and Strauss [31]. A total of approximately 8 to 12 interviews per country were sufficient to capture both the breadth and depth of relevant insights.

The process of recruiting participants for interviews was initiated on August 1, 2024, and has been concluded by September 30, 2025. The initial contact was made via email. The email contained a brief overview of the project and an offer to meet online to discuss any queries. Responses were followed up, and where no response was received, a follow-up email was sent, and in some cases, telephone contact was made. Attempts were made to arrange further interview appointments through existing contacts in the country. The recruitment period for each country commenced approximately 2 to 3 months before the onsite data collection. Before the interview, the participants receive information on the general conditions of the interviews. This includes the aim of the project, the type and duration of the interview, and the further use of the data. Consent was also obtained from the interviewees to make audio recordings and to ensure later transcription and analysis of the data.

#### Collecting and Analyzing the Interview Data

**Figure 4.**
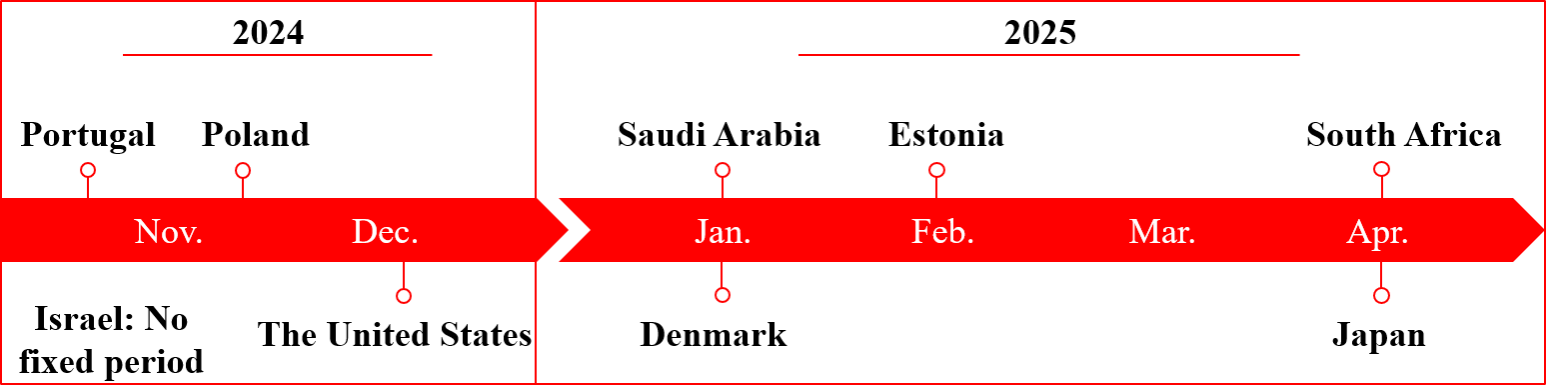
Schedule of the interviews. This figure shows the schedule for the expert interviews, which are conducted onsite from November 2024 to April 2025.

A team of 4 experienced researchers (LK, ALB, SS, and VEA) conducted individual interviews with experts in 8 of the 9 countries on site from November 2024 to April 2025. Further interviews were carried out remotely. The data collection for all case studies has been completed by the end of September 2025. A total of more than 100 expert interviews were conducted. The schedule of the interviews can be seen in Figure 4. In Israel, the interviews were conducted remotely over the entire survey period until the end of September 2025. If an onsite appointment at the workplace of the expert or a public place was not feasible, an online interview was arranged. The approach to the interviews was based on the specially created semistructured interview guide. The interviews are scheduled for 30 to 45 minutes. Both the onsite interviews and the digital conversations were recorded by an appropriate recording device. The audio recordings were transcribed with the support of artificial intelligence within the software MAXQDA Transcription (VERBI Software GmbH) and the integrated transcription function in Microsoft Word. The transcriptions are revised in terms of content and formatting. The transcription is based on the content-semantic transcription according to Dresing and Pehl [27]. Consequently, citations are not made according to spoken language, but verbally. Furthermore, time stamps are set, and the paragraphs are divided by interviewer and expert. The evaluation of the data within the finished transcripts is carried out manually within the MAXQDA software using the qualitative content analysis according to Kuckartz and Rädiker [28]. In accordance with the procedure of qualitative content analysis, inductive and deductive coding is carried out by researchers within the framework of a category system. Deductive codes are developed based on the semistructured interview guide, which covers topics such as governance, incentive mechanisms, and interoperability. Inductively, researchers primarily add subcategories that are assigned to the main codes. This qualitative content analysis will enable them to evaluate interviews based on relevant criteria, such as success factors or tipping points. Consequently, the analysis will yield hypotheses about possible success factors, the transfer of which will be evaluated in a subsequent quantitative survey.

### Quantitative Survey

In an iterative process, the quantitative survey will be carried out based on the results of the qualitative interviews. The aim of this cross-sectional data collection is to assess the transferability of key findings from the case studies to the context and status quo of digital health in Germany. Furthermore, it will be analyzed which successful strategies and measures, observed in other health care systems, could be implemented in the German system. To this end, a customized online questionnaire will be developed based on the qualitative data obtained. A set of closed and semi-open questions will be designed. The developed questionnaire will be pretested in a 2-step procedure. In the first step, the wording of the questions and the response categories will be checked in an internal pretest with 2 to 3 methodological experts. If required, the questionnaire will then be optimized as necessary. In the second step, the adapted questionnaire will be field-tested in the targeted population, with a sample of at least 10 participants, with each subgroup being represented by 2 people [29]. The period for recruitment is scheduled to take place from October 15 to December 15, 2025. The survey instrument will be disseminated in various iterations to relevant stakeholder groups within the German health care system, following a convenience sampling approach, including patient representatives, health insurance funds, associations of statutory health insurance, physicians, professional organizations, and other stakeholders in health care policy. The online questionnaire will be distributed via email, social media, and professional networks. Before completing the questionnaire, participants are informed about data collection, data protection, and data confidentiality, and consent is obtained.

The required minimum sample size to achieve adequate statistical power was calculated a priori using the software program G*Power [31]. To identify differences and correlations between the 5 subgroups of a medium effect size with 95% power, a sample size of a minimum of 305 participants is required, equating to 60 participants per stakeholder group. The objective is to obtain a response rate of minimum of a minimum of 305 to 400 participants.

The collected questionnaires are subjected to a process of review, during which the answers will be checked for completeness and plausibility. If necessary, data cleansing will be performed according to predefined criteria. The final quantitative data will be analyzed using the statistical analysis software IBM SPSS (version 30.0.0). Therefore, both descriptive and inferential tests will be used. For the descriptive analysis, measures of absolute and relative frequencies, central tendency (mode, median), and dispersion (range, IQR) will be reported. Correlations and subgroup differences will primarily be analyzed using the Spearman rank correlation and the Kruskal-Wallis test. Finally, the key results of the quantitative evaluation will inform the development of recommendations for action.

### Ethical Considerations

Before the initiation of the study, a data protection concept was formulated, encompassing the methodology for data collection and the protocol for data protection, as delineated by the researchers. A consent form was developed as part of the data protection concept and presented to each participant in an expert interview. By signing this consent form, the interviewer and interviewee agreed to the processing of the data. The audio files and transcripts are accessible exclusively to the project team at Hannover Medical School. The results of the qualitative content analysis will be disseminated without identification of the interview partners. Participants in the quantitative survey were also informed about data collection and privacy, and consent was obtained before participation in the survey as a mandatory part of participation. The collection and analysis of data are conducted anonymously. Subsequent to the formulation of this concept, the ethics vote of the ethics committee of Hannover Medical School, Ethikkommission der Medizinischen Hochschule Hannover, was obtained. The study approach was approved by the members of the ethics committee (approval number: 11440_BO_S_2024) and was deemed to be “without any concerns.” The study procedure is consistently aligned with the Declaration of Helsinki and the principles of good clinical practice [32]. The interviewees and participants in the survey did not receive any compensation.

## Results

The data collection process for our qualitative research method was initiated in November 2024 and completed by the end of September 2025. A total of 109 expert interviews were conducted. The analysis and interpretation of the data will be completed in December 2025. The initial results of the qualitative survey are targeted for publication in spring 2026. In October 2025, the online questionnaire was disseminated to the relevant stakeholders. The analysis of the quantitative data and its subsequent submission for publication are scheduled for 2026.

## Discussion

### Anticipated Findings

In light of the challenges facing the digital transformation of the German health care system, as outlined in the introduction, the focus of this research project is particularly timely and relevant. Despite recent progress in digital health, Germany continues to face significant barriers that hinder the full implementation and realization of digital health’s potential benefits. By systematically analyzing the success factors behind the digitalization efforts of other health care systems, the results of this research project will contribute to the development of evidence-based policy recommendations for the digital transformation of health care systems in Germany. By integrating insights from qualitative expert interviews and quantitative survey data, the study provides a comprehensive understanding of the political, social, and technological factors driving digitalization. This approach will help identify key success factors and address existing research gaps in the national context. Ultimately, the findings will support tailored recommendations that not only advance Germany’s health care policies but also enhance its competitiveness at an international level.

In the following, the strengths and limitations of the methodological approach, the selection of the case studies, as well as the qualitative and quantitative survey are discussed.

### Scope

This study adopts a broad perspective on the digital transformation of health care systems, encompassing various health care systems as case studies and incorporating insights from diverse stakeholders and experts across different countries. Unlike research that focuses on specific technological innovations or applications, this study examines the political and social processes that shape digital transformation in health care. The advantage of this approach is its ability to generate comprehensive insights and provide a holistic understanding of a highly complex topic. However, a significant challenge is ensuring that all relevant perspectives and aspects are adequately captured, analyzed, and interpreted, given the multitude of factors influencing digitalization processes in different health care systems.

### Methodological Approach

The study uses a mixed methods approach, which strengthens the validity and robustness of the findings. Combining qualitative and quantitative methods allows the research to benefit from the in-depth understanding generated through semistructured expert interviews and to obtain more generalizable results through the quantitative online survey. The qualitative component of this approach is based on case study research. While case studies are sometimes criticized for lacking systematic structure and methodological flexibility, they are well suited to addressing the research questions posed in this study. The flexibility intrinsic to case study research enables an in-depth exploration of contextual factors and dynamics within each health care system, providing rich insights that would be difficult to obtain through more rigid methodological frameworks. To enhance the reliability and objectivity of the findings, this study follows the recommendations of Merriam [11], ensuring a structured approach to case study research and mitigating potential biases. The quantitative survey, in turn, provides a broader empirical foundation to validate these insights, particularly in the context of the German health care system.

The selection of case study countries posed a considerable challenge. In other studies, the selection of cases is often presented in a nontransparent or overly concise manner. In contrast, this study is based on a targeted selection process grounded in extensive research and discussions with experts in the field. The primary difficulty lies in accounting for the numerous criteria that influence the success factors of digital transformation within a health care system and evaluating the potential for the transfer of these factors to the German health care system. In addition, the selection process must account for limited human and financial resources available. The diverse case selection approach used in this study is well suited for exploratory research [12]. By selecting countries that exhibit a broad range of characteristics in terms of geographical region and size, economic power, health care system typology, and governance structure, this approach ensures a wide spectrum of insights into digital transformation processes. While this method does not prioritize representativeness, it allows for a more nuanced understanding of the diversity of digital health strategies and facilitates the identification of relevant success factors that may inform digital health strategies in Germany. Despite the inherent challenges, the selection of case studies is appropriate for addressing the research questions and maximizing knowledge gain.

### Qualitative Survey

The data collection within the case studies was carried out using a qualitative methodology based on expert interviews. The choice of this qualitative method proved to be suitable as it enabled the researchers to gain in-depth and comprehensive insights into the experiences and perspectives of the experts in the field of digital health.

The selection of experts was made on the basis of their demonstrated proficiency in the domain of digital health, with the objective of acquiring particular insights and experiences during the course of the interviews. Furthermore, given the heterogeneity of the occupational distribution of the interviewees, it was anticipated that a broad spectrum of perspectives would be obtained. Accordingly, the interviewees work in the areas of health care policy, payers, service provision (outpatient and inpatient), IT and the industrial sector, patient representation, science, and beyond. The comprehensive perspectives incorporated in this study are a notable strength. The number of interviewees per health care system was set at a minimum of 8 to 12, with the intention of attaining a state of saturation. However, due to the unavailability of some interviewees during the specified period, alternative dates were arranged for the remaining interviews to be conducted remotely. The researchers also encountered challenges in recruiting participants due to cultural differences in scheduling appointments and infrequent feedback from the experts.

The use of a structured interview guide was instrumental in facilitating the systematic conduct of the interviews, thereby ensuring consistency in the formulation of pertinent inquiries by the researchers. This systematic approach was meticulously designed to guarantee the exploration of specific key topics. The use of a semistructured design was deliberate, with the objective being to capture the diverse perspectives of the experts.

The majority of the interviews were planned as face-to-face conversations with the experts on site. This allows the researchers to get a first impression of the digital conditions in the country and in different institutions. The advantage of conducting interviews in person is that nonverbal signals, such as body language, tone of voice, and gestures, can be captured. However, some of the interviews were carried out in a location with pronounced background noise, which made transcription difficult, although not impossible largely due to the use of AI transcription tools. The addition of remote interviews was also extremely useful to interview experts who were difficult to reach. However, it should be noted that remote interviews are susceptible to technical challenges, including unstable internet connections and poor audio quality. Nonetheless, with adequate preparation, these issues can be mitigated.

Concerning the evaluation of results, it is important to acknowledge the inherent subjectivity of researchers [27]. The management of data, its categorization, and the allocation of locations may consequently yield a distorted representation of the data. Ensuring the analysis is conducted by 2 researchers and that there is a regular exchange of information on the status of the evaluation to date can ensure intersubjective traceability and achieve an equal understanding of the analysis process. In addition, the findings will be presented transparently [27].

### Quantitative Survey

As part of the planned quantitative survey, within which the findings from the case studies are to be tested for the German health care system, it is essential to create a customized questionnaire based on the qualitative preliminary work. This will enable the analysis of relevant success factors. However, the challenge here is to precisely operationalize complex concepts, such as digital acceptance and implementation barriers, so that they remain measurable and interpretable.

Another challenge could lie in recruiting a sufficiently large and diversified sample to enable reliable statements for different stakeholder groups. The aim is to achieve a sample size of 305 to 400 completed questionnaires, and it is acknowledged that self-selection could lead to bias, as people with a strong interest in the topic could participate. This should be counteracted by a broad distribution of the online questionnaire. It should also be mentioned that possible limitations in data analysis due to sample selection bias or nonresponse bias must be taken into account. The statistical analysis, which is to be conducted using the statistical analysis software SPSS, will enable a systematic evaluation.

Following the analysis of the quantitative survey, the core results that can be transferred to the German health care system will be determined, as well as the extent and form in which they can be transferred. This issue will be tackled with a clearly defined plan that forms the basis of the research project and is regularly reviewed through regular dialogue within the research team and with the project’s cooperation partners.

### Conclusions

In summary, the integration of qualitative case studies and quantitative transferability assessment within a mixed methods approach is expected to generate valuable insights into the key factors and tipping points that influence the successful implementation of digital health care solutions. In addition, all relevant stakeholders in the health care sector can be considered comprehensively. The insights gained from this study will not only shed light on the success factors that have fundamentally contributed to digital transformation in international health care systems. They will also inform effective recommendations for action to accelerate the digital transformation in the German health care system.

## Supplementary material

10.2196/80301 Multimedia Appendix 1 Shortlist of countries considered for case study selection and their key characteristics.

10.2196/80301 Multimedia Appendix 2 Semistructured interview guide.

10.2196/80301 Checklist 1 COREQ checklist.

## Abbreviations

COREQ: Consolidated Criteria for Reporting Qualitative Research
