# Supplementary material for: International Case Studies to Identify Success Factors and Contextual Conditions in the Digital Transformation of Health Care Systems and Derive Lessons for Germany: Study Protocol for a Mixed Methods Study
Source: JMIR Res Protoc. 2026 Jan 21;15:e80301. doi: 10.2196/80301 (PMC12822856; doi:10.2196/80301)
Supplement: Multimedia Appendix 1 [file resprot-v15-e80301-s001.docx]

**Supplement 1: Shortlist of Countries Considered for Case Study Selection and Their Key Characteristics**

| Country | Level of General Digitalisation^1^ | Geography | | | Economy | | Healthcare System | | |
| --- | --- | --- | --- | --- | --- | --- | --- | --- | --- |
|  |  | **WHO-Region** | **Population**  (in million people,  2023 or l.a.^2^) | **Area**  (in km^2^) | **Economic Development** | **Economic Performance**  (GDP/capita in US$, current prices,  2022 or l.a.) | **Healthcare System Typology**^3^ | **Health Expenditure**  (in US$/capita, current prices, 2022 or l.a.) | **Centralisation of Healthcare Governance**^3^ |
| Algeria | Emerging | African Region | 44.9 | 2,381,741 | Developing | 4,962 | National Health Service | 180 | Central/Decentral |
| South Africa | Emerging |  | 59.9 | 1,213,090 | Emerging | 6,523 | Social Insurance/  Private Insurance | 570 | Decentral |
| Tanzania | Emerging |  | 65.5 | 885,800 | Developing | 1,208 | National Health Service/ Private Insurance | 36 | Central/Decentral |
| Egypt | Emerging | Eastern Mediterranean Region | 110.10 | 995,450 | Emerging | 4,233 | National Health Service | 171 | Decentral |
| Saudi Arabia | Emerging |  | 36.4 | 2,149,690 | Emerging | 34,454 | National Health Service/  Private Insurance | 1,593 | Central |
| United Arabian Emirates | Advanced/  Emerging |  | 9.4 | 71,020 | Emerging | 49,899 | National Health Service/Private Insurance | 2,352 | Decentral |
| Denmark | Advanced | European Region  European Region | 5.9 | 41,987 | Developed | 68,091 | National Health Service | 6,456 | Decentral |
| Estonia | Advanced |  | 1.4 | 43,110 | Developed | 28,451 | Social Insurance | 1,999 | Central |
| Finland | Advanced |  | 5.6 | 303,948 | Developed | 50,439 | National Health Services | 4,902 | Decentral |
| Israel | Advanced |  | 9.8 | 21,640 | Developed | 54,931 | Social Insurance | 4,224 | Central |
| Netherlands | Advanced |  | 17.7 | 33,670 | Developed | 59,123 | Social Insurance/Private Insurance | 5,796 | Decentral |
| Poland | Emerging |  | 36.6 | 307,236 | Developed | 18,891 | Social Insurance | 1,193 | Central |
| Portugal | Emerging |  | 10.6 | 90,977 | Developed | 24,621 | National Health Service | 2,581 | Central |
| Sweden | Advanced |  | 10.5 | 407,300 | Developed | 55,297 | National Health Services | 5,943 | Decentral |
| Switzerland | Advanced |  | 8.9 | 39,510 | Developed | 93,249 | Social Insurance/  Private Insurance | 10,963 | Decentral |
| Canada | Advanced | Region of the Americas | 38.9 | 9,093,507 | Developed | 55,509 | National Health Service | 6,255 | Decentral |
| Dominican Republic | Emerging |  | 111.2 | 47,531 | Emerging | 10,110 | Social Insurance/  Private Insurance | 462 | Central |
| United States of America | Advanced |  | 333.3 | 9,147,420 | Developed | 78,035 | Private Insurance | 12,434 | Decentral |
| Cambodia | Emerging | South-East Asian Region | 16.8 | 176,520 | Developing | 2,325 | Privat Insurance/  Social Insurance | 110 | Central |
| India | Emerging |  | 1420.0 | 2,973,190 | Emerging | 2,353 | Social Insurance/ Privat Insurance/ | 80 | Decentral |
| Indonesia | Emerging |  | 275.5 | 1,892,555 | Emerging | 4,731 | Privat Insurance/  Social Insurance | 127 | Central |
| Japan | Advanced | Western Pacific Region  Western Pacific Region | 1393.4 | 364,500 | Developed | 34,017 | Social Insurance | 3,889 | Central |
| South Korea | Advanced |  | 51.6 | 97,600 | Developed | 32,395 | Social Insurance/  Private Insurance | 3,050 | Central |
| Singapore | Advanced |  | 5.6 | 718 | Developed | 88,429 | Social Insurance/  Private Insurance | 4,321 | Central |

Note: Selected case studies are highlighted in grey.

^1^ Classification is based on international digitalization rankings (see references) as well as expert opinion.

^2^ latest available

^3^ Classification is based on literature research (see references).

**References:**

1. Alsamara T, Farouk G, Mallaoui H. Administrative Organization of Health Care Institutions in Algeria: Between Centralization and Decentralization. Open Acces Macedonian Journal of Medical Science 2022; 10(E):1114-1118.
2. Asia Pacific Observatory on Health Systems and Policies. The Republic of Indonesia. Health System Review. Health Systems in Transition 2017; 7(1). Available from: URL: https://iris.who.int/bitstream/handle/10665/254716/9789290225164-eng.pdf;sequence=1.
3. European Center for Digital Competitiveness. Digital Riser Report 2020; 2020. Available from: URL: https://digital-competitiveness.eu/#studies
4. European Center for Digital Competitiveness. Digital Riser Report 2021; 2021. Available from: URL: https://digital-competitiveness.eu/digitalriser/.
5. European Commission. Digital Economy and Society Index (DESI) 2022; 2022. Available from: URL: https://digital-strategy.ec.europa.eu/en/library/digital-economy-and-society-index-desi-2022.
6. Federal Office for Migration and Refugees. Länderreport 45 Tansania. Allgemeine Situation und Menschenrechtslage; 2021. Available from: URL: https://www.bamf.de/SharedDocs/Anlagen/DE/Behoerde/Informationszentrum/Laenderreporte/2021/laenderreport-45-Tansania.pdf?__blob=publicationFile&v=2.
7. Federal Statistical Office (Destatis). Country Profiles; 2024. Available from: URL: https://www.destatis.de/DE/Themen/Laender-Regionen/Internationales/_inhalt.html#_43q9s9d00.
8. IMD World Competitiveness Center. IMD World Digital Competitiveness: Ranking 2023; 2023. Available from: URL: https://www.imd.org/centers/wcc/world-competitiveness-center/rankings/world-digital-competitiveness-ranking/.
9. Koornneef E, Robben P, Blair I. Progress and outcomes of health systems reform in the United Arab Emirates: a systematic review. BMC Health Services Research 2017; 17:672.
10. Tikkanen R, Osborn R, Mossialos E, Djordjevic A, Wharton G (The Commonwealth Fund, London School of Economics and Political Science). International Profiles of Health Care Systems; 2020. Available from: URL: https://www.commonwealthfund.org/sites/default/files/2020-12/International_Profiles_of_Health_Care_Systems_Dec2020.pdf.
11. World Bank Group. Data Bank. World Development Indicators; 2025. Available from: URL: https://databank.worldbank.org/source/world-development-indicators/preview/on.
12. World Health Organization, European Observatory on Health Systems and Policies. Health Systems in Action. Israel; 2024. Available from: URL: https://iris.who.int/bitstream/handle/10665/380238/9789289059749-eng.pdf?sequence=1.
13. World Health Organization. Global Health Expenditure Database; 2025. Available from: URL: https://apps.who.int/nha/database.
