# Supplementary material for: International Case Studies to Identify Success Factors and Contextual Conditions in the Digital Transformation of Health Care Systems and Derive Lessons for Germany: Study Protocol for a Mixed Methods Study
Source: JMIR Res Protoc. 2026 Jan 21;15:e80301. doi: 10.2196/80301 (PMC12822856; doi:10.2196/80301)
Supplement: Multimedia Appendix 2 [file resprot-v15-e80301-s002.pdf]

<table>
<tr><th colspan="3">Semi-Structured Interview Guide</th></tr>
<tr><td colspan="3">Introduction to the conversation
Introduction of the interviewer
Consent form
Presentation of the topic
Academic research project on the implementation of digitalization in healthcare
International Case Studies in nine different countries
Interviews with experts in digital health
Procedure of the interview
30-minutes
Recording for transcription later
Natural conversation
<u>Please only answer specifically for country XX in the entire interview.</u>
[Note that recording is starting.]</td></tr>
<tr><th colspan="3">Narrative-generating introductory question</th></tr>
<tr><td colspan="3">Entry question

Today we would like to talk about the digital transformation of the healthcare system in country XX with you and explore what truly made the digital health transformation in country XX take off.

What sparked your interest in digital health, and how did you find yourself working in this area?</td></tr>
<tr><th>Central Question</th><th>Checklist –
Have these points been mentioned?</th><th>Further questions</th></tr>
<tr><th colspan="3">Module I: Tipping Points
"Tipping points" refer to pivotal moments when changes trigger a significant and often irreversible shift in a system. In the context of implementing digital health tools, tipping points occur when certain changes lead to widespread adoption and fundamental changes in healthcare delivery.</th></tr>
<tr><td>Personal Perspective on Digital Health Transformation

From your perspective as XX, how would you describe the digital health transformation in country XX?</td><td>Personal experience
Implementation of digital health tools</td><td>In your opinion, what went well (or badly) when implementing digital health tools?

What were the success factors in implementing digital applications to your everyday work?

[HCP]: How have you been able to integrate digital tools into your everyday work?</td></tr>
<tr><td>Past Tipping Points

Which events have driven the digital health transformation in country XX the most?</td><td>Influence of important events
Decision makers
Long-term strategies
Legal reforms</td><td>Why did these events lead to this transformation or acceleration of digitalization?

Which long-term strategies have driven digitalization the most and why?

What potential opportunities for digital health transformation have been missed or not optimally utilized?</td></tr>
</table>

**Current Developments and future Tipping Points**<br><br>Are there current developments that could influence the future digital health transformation in *country XX*?	• Current trends<br>• Expected events<br>• New strategies	What trends or changes do you expect in the digital health transformation in *country XX*?<br><br>Are there any specific technologies that you expect to see more widely implemented in the next few years in *country XX* and why do you think they will be implemented broadly?
**Module II: Governance**		
How do you perceive the impact of the healthcare system's structure (e.g. typology, influence of various stakeholders) on the digital health transformation in *country XX*?<br><br>Which stakeholders have driven the digital transformation/the implementation of digital health tools the most and how would you describe the influence/role of other relevant stakeholders in this process?	• Top-down vs. Bottom-up<br>• Shared Decision<br>• Integration	Have there been any recent changes in the governance structure of the healthcare system in *country XX* and how did they affect the digital health transformation?<br><br>How is the leadership regarding the implementation of digital health tools structured? (top-down vs. bottom-up)<br><br>How did you/your organization become involved in the political process?
**Module III: Incentives and sanctions**		
**Influence of incentives**<br><br>Which incentive mechanisms had an impact on the digital transformation *(in your role)*?	• Quality<br>• Patient-centered<br>• Care<br>• Recognition<br>• Opportunities for advancement<br>• Autonomy	Could you explain why they had an impact?<br><br>Which incentive mechanisms do you consider crucial to ensure a successful and sustainable digital transformation in the long term?
**Monetary Incentives**<br><br>*[HCP]:* In what manner have financial incentives or government funding programs had an influence on the implementation or use of digital technologies in your work?<br><br>*[IT/Industry/Payer]:* In what manner have market mechanisms in *country XX* had an impact on the digital transformation?	• Installment payments<br>• Fines<br>• Based on number of entities	*[HCP]:* Has the introduction of digital technologies changed the way you are reimbursed for your services?<br><br>*[HCP]:* Have you experienced financial incentives blocking or slowing down the process of digital transformation?<br><br>*[HCP]:* In what way did the financing system have an influence on digital transformation in your practice?<br><br>*[IT/Industry/Payer]:* Have your business models had an impact on the digital transformation?

**Influence of sanctions**<br><br>In which way have sanctions mechanisms in *country XX* had an influence on the implementation or use of digital technologies in your work?	• Penalties	Could you tell us more about the sanctions that have or had an impact on the digital transformation in your work?<br><br>Do you have any ideas on how sanctions could be designed to ensure that the use of digital technologies continues in the long term and not just achieve short-term goals?
**Module IV: Technical regulations**		
**Health information exchange infrastructure**<br><br>How is the infrastructure for health data exchange mechanisms in *country XX* organized and how does this affect the digital health transformation?	• Influence of infrastructure on digital health transformation	How efficient is the health information exchange infrastructure mechanisms in *country XX*? / How well does the health information exchange system work mechanisms in *country XX*?<br><br>Have there been any events in recent years that have had an impact on the implementation of the health information exchange infrastructure mechanisms in *country XX*?<br><br>How does the health information exchange infrastructure impact the implementation of new digital health tools?
***Interoperability** in healthcare refers to the ability of different systems and technologies to exchange information and use that information effectively. This enables seamless communication between various healthcare providers, software applications, and devices, allowing for efficient and secure transfer of patient data among different stakeholders in the healthcare system.*		
**Interoperability**<br><br>How is healthcare interoperability organized in *country XX* and how efficient are these structures?	• Responsibilities<br>• Standards	To what extent are interoperability requirements implemented?<br><br>How important is the interoperability of IT systems and digital applications for the success of digital health transformation?<br><br>What are the success and failure factors in the organization of interoperability in *country XX*?<br><br>Which organizations are involved in the development of standards for interoperability and how important are they?<br><br>What effect do international (e.g. EHDS), or national interoperability

		standards have on digital health transformation? Do they tend to hinder or promote digitalization?<br><br>What technological or regulatory improvements would be necessary to further strengthen interoperability?
**Module V: Digital Health Tools**<br>[2 Tools per Expert]<br>*Digital Health Tools:* ***Concentric Value Model****: Value influence of Digital Health Tools on the dimensions of subject, interaction, system and society*		
**System-oriented tools**<br>*[EHR, electronic prescription, etc.]*<br><br>*[HCP]:* Do you use system-oriented tools?<br><br>*[HCP/Payer/Patient Representatives/Politics/Science]:* How was the implementation process of system-oriented tools perceived and what were the challenges during the implementation?<br><br>*What influence does the digital technology have on different dimensions, so users, the interaction of users, the healthcare system and society as a whole?*	• Implementation process<br>• Support<br>• Challenges<br>• *CVM: Perspective on the value influence on the dimensions of subject, interaction, system and society*	Which circumstances were most important for the successful implementation?<br>- *This means that the introduction of the use of this tool*<br><br>Can you explain whether and, if so, to what extent the use of the tool has had a positive impact on your work?<br><br>*[Industry/IT]:* How do you ensure that your tools have a positive impact on healthcare delivery?
**Tools to support health care professionals in optimizing collaboration with each other**<br>*[This means applications such as telemedicine, digital documentation and data management]*<br><br>*[HCP]:* Do you use tools to optimize collaboration with other HCPs?<br><br>*[HCP]:* How did the implementation process take place, how did you perceive it and what were the challenges during implementation?	• Implementation process<br>• Support<br>• Challenges	Which circumstances were most important for the successful implementation?<br><br>Did the use of the tool have a positive impact on your work?

<table>
<tr><td>Prevention-oriented tools

[HCP]: Do you use prevention-oriented instruments for the promotion of the health of your patients?

IT/Industry/Payer/Science: How was the implementation process of prevention-oriented tools perceived and what were the challenges during the implementation?</td><td>Implementation process
Support
Challenges</td><td>Which circumstances were most important for the successful implementation?</td></tr>
<tr><td>Indication-oriented tools
[e.g. for the support of patients with chronic diseases]

[HCP/IT/Industry /Patient Representatives/Payer/Science]: How was the implementation process of indication-oriented tools perceived and what were the challenges during the implementation?</td><td>Implementation process
Support
Challenges</td><td>Which circumstances were most important for the successful implementation?</td></tr>
<tr><th colspan="3">Module VI: Closing questions</th></tr>
<tr><td>Future transformation

What are key lessons in the digitalization of a healthcare system?</td><td>Actions that need to be taken
Key lessons</td><td>Ask for further explanation if the point has not become clear.

No further questions necessary.</td></tr>
<tr><td>Additions by interviewee

Is there anything else we forgot that you would like to address?</td><td>Open question, so as not to influence the form of the answer</td><td>No further questions necessary.</td></tr>
<tr><td colspan="3">End of the Interview
[Note that recording is finished.]
Thank the interviewee for participating and for their willingness to provide information.</td></tr>
</table>
